# Supplementary material for: Content-rich biological network constructed by mining PubMed abstracts
Source: BMC Bioinformatics. 2004 Oct 8;5:147. doi: 10.1186/1471-2105-5-147 (PMC528731; doi:10.1186/1471-2105-5-147)
Supplement: Additional File 2 — The original results of the above study (non-essential files are deleted to keep the file size under the limit set by BMC bioinformatics). [file 1471-2105-5-147-S2.bz2 › chilibotAdditionalFile2/dip30/161ID8612709E566/html/ACVR1_ACVR2.html]

 


 **ACVR1** and **ACVR2** 
  
Found 63 abstracts in PubMed, retrieved 30.  
 

 What does Google say? 
 PDF only 
| .edu only 

---

**Interactive relationship** (e.g. stimulation, inhibition, etc)

**Non-interactive relationship** (e.g. studied together, co-existance, homology, etc.)

- Activin A bound to ActR IB ALK 4 and ActR II  [ **ACVR2** ] , and BMP 7 bound to ActR I  [ **ACVR1** ]  ALK 2  [ **ACVR1** ] , BMP type I receptor BMPR I ALK 3, ActR II  [ **ACVR2** ]  and BMPR II.  Ref: 10574705 J Cell Sci, 1999
- BMP 6 strongly bound to activin receptor  [ **ACVR2** ]  like kinase ALK 2  [ **ACVR1** ]  also termed ActR I  [ **ACVR1** ] , together with type II receptors, i.e. BMP type II receptor BMPR II and activin type II receptor ActR II  [ **ACVR2** ] .  Ref: 10504300 J Cell Sci, 1999
- Both type I and type II activin receptor  [ **ACVR2** ]  ActR I  [ **ACVR1** ] , ActR IB and ActR II  [ **ACVR2** ]  mRNA expression was also detectable in the pheochromocytoma tissues.  Ref: 10810314 J Endocrinol, 2000
- To investigate the expression and function of specific Type I TGFbeta receptors during AV cushion transformation, we cloned and characterized the chicken homologues of two mammalian activin receptor  [ **ACVR2** ]  like kinases ALK, ALK2  [ **ACVR1** ]  and ALK5, and generated specific, polyclonal antibodies against the extracellular binding domains of each.  Ref: 10885742 Dev Biol, 2000
- A mechanistic explanation for increased pSmad1 5 staining during inflammation was provided by the upregulated expression of all the BMP type I receptors, i.e., activin receptor  [ **ACVR2** ]  like kinase ALK 2  [ **ACVR1** ] , ALK3, and ALK6, in the inflamed bronchial epithelial cells.  Ref: 12151307 Am J Respir Cell Mol Biol, 2002
- The type I receptors examined were the TGF beta type I receptor TbetaR I, activin type I receptor  [ **ACVR1** ]  ActR I  [ **ACVR1** ] , activin type IB receptor ActR IB, bone BMP type IA receptor BMPR IA, BMP type IB receptor BMPR IB, and activin receptor  [ **ACVR2** ]  like kinase 1 ALK 1.  Ref: 10321529 Acta Ophthalmol Scand, 1999
- The ActR I  [ **ACVR1** ]  activin receptor  [ **ACVR2** ]  protein is expressed in notochord, lens placode and pituitary primordium cells in the mouse embryo.  Ref: 10704880 Mech Dev, 2000
- BMP type II receptor BMPR II is a type II serine threonine kinase receptor, which transduces signals for BMPs through heteromeric complexes with type I receptors, including activin receptor  [ **ACVR2** ]  like kinase 2  [ **ACVR1** ]  ALK2  [ **ACVR1** ] , ALK3 BMPR IA, and ALK6 BMPR IB.  Ref: 10772805 Dev Biol, 2000
- When coexpressed with type I activin receptor  [ **ACVR2** ]  ActR I  [ **ACVR1** ]  or ActR IB in concert with type II receptor ActR II  [ **ACVR2** ] , Smad7 efficiently reduced an early transcriptional response mediated by ActR I  [ **ACVR1** ]  but had only a minimal effect on the response mediated by ActR IB.  Ref: 10828018 Blood, 2000
- these cells were found to express mRNA for BMP receptors including, ActR I  [ **ACVR1** ] , BMPR IA, ActR II  [ **ACVR2** ] , ActR IIB, and BMPR II.  Ref: 11786084 Kidney Int, 2002
- RESULTS Activin A and receptors ActR I  [ **ACVR1** ] , ActR IB, ActR II  [ **ACVR2** ]  are transcribed in corneal fibroblasts.  Ref: 11773015 Invest Ophthalmol Vis Sci, 2002
- PCR products with the expected sizes for ActR I  [ **ACVR1** ] , ActR IB, ActR II  [ **ACVR2** ] , and ActR IIB mRNAs were detected in freshly dissociated and 5 day cultured granulosa luteal cells.  Ref: 10210281 Life Sci, 1999
- We have also explored if there are associated changes in activin receptor  [ **ACVR2** ]  Alk2  [ **ACVR1** ] , ActRII  [ **ACVR2** ]  and ActRIIB in these tissues.  Ref: 11572790 J Endocrinol, 2001
- Furthermore, transfection with wild type activin receptor  [ **ACVR2** ]  type I  [ **ACVR1** ]  results in activin mediated suppression of cell proliferation.  Ref: 12364468 J Clin Endocrinol Metab, 2002
- Constitutively active forms of BMP type I receptors BMPR IA and BMPR IB and those of activin receptor  [ **ACVR2** ]  like kinase ALK 1 and ALK 2  [ **ACVR1** ]  maintained differentiation of chondrocytes in the presence of retinoic acid.  Ref: 12565908 Biochem Biophys Res Commun, 2003
- Activin receptor  [ **ACVR2** ]  like kinase 2  [ **ACVR1** ]  can mediate atrioventricular cushion transformation.  Ref: 10885742 Dev Biol, 2000
- Three lysines, Lys 431, Lys 440, and Lys 441 in p50 were all acetylated in vitro, and a sequence similarity among p50, p53, Tat, and activin receptor  [ **ACVR2** ]  type I  [ **ACVR1** ]  on these particular lysines was observed.  Ref: 11739381 J Biol Chem, 2002
- We then investigated whether the sensitivity of the early long bone rudiments to OP 1 correlated with high expression of the OP 1 binding type I serine threonine kinase receptors activin receptor  [ **ACVR2** ]  like kinase ALK 2  [ **ACVR1** ]  ActR I  [ **ACVR1** ] , ALK 3 BMPR IA or ALK 6 BMPR IB at this early stage.  Ref: 10705576 Growth Factors, 2000
- The presence of inhibin subunits alpha, betaA and betaB, activin binding protein follistatin and activin receptors ActR I  [ **ACVR1** ] , ActR IB, ActR II  [ **ACVR2** ]  and ActR IIB mRNA transcripts in bovine oocytes and preimplantation embryos was examined by reverse transcription polymerase chain reaction.  Ref: 11596877 Reprod Fertil Dev, 1998
- Analysis in native tissues from 12 normal thyroids, 16 goiters, and 5 Graves thyroids demonstrated mRNA expression of the activin subunit betaA, TGFbeta receptor type I and II, activin receptors type I receptors alk2  [ **ACVR1** ]  and alk4, and activin type II receptors actRII  [ **ACVR2** ]  and actRIIb.  Ref: 11069203 Horm Metab Res, 2000
- Furthermore, bovine oocytes and embryos express the activin receptors mainly by the combination of ActR I  [ **ACVR1** ]  and either ActR II  [ **ACVR2** ]  or ActR IIB.  Ref: 11596877 Reprod Fertil Dev, 1998
- Primers specific for two type I and two type II activin receptors ActR I  [ **ACVR1** ] , ActR IB, ActR II  [ **ACVR2** ] , and ActR IIB were used in polymerase chain reaction PCR to amplify cDNAs prepared from granulosa luteal cells, placental tissues and isolated trophoblast cells.  Ref: 10210281 Life Sci, 1999
- There were no apparent differences in the distribution of the beta A subunit and receptors Alk2  [ **ACVR1** ] , ActRII  [ **ACVR2** ]  and ActRIIB between control and preeclamptic tissues.  Ref: 11572790 J Endocrinol, 2001
- Activin receptor  [ **ACVR2** ]  like kinase ALK 1, ALK 2  [ **ACVR1** ] , ALK 3, and ALK 6 were able to reproduce the hypertrophic maturation of chondrocytes induced by BMP 2.  Ref: 12082094 J Biol Chem, 2002
- Receptor Alk2  [ **ACVR1** ]  protein levels were significantly elevated in T PE placentae P=0.0006, Mann Whitney U test, ActRIIB levels were significantly lower in PT PE placentae P=0.01 and ActRII  [ **ACVR2** ]  levels were significantly lower in PT PE choriodecidua P=0.0002 compared with controls.  Ref: 11572790 J Endocrinol, 2001
- In this study, we found the expression of activin type I receptors ActR I  [ **ACVR1** ]  and ActR IB and type II receptor ActR II  [ **ACVR2** ]  on human fetal lung fibroblasts HFL 1 by RT PCR and immunocytochemistry.  Ref: 11261590 Life Sci, 2000
- Renal expression of the high affinity BMP type II receptor and the type I receptor Alk2  [ **ACVR1** ]  activin receptor  [ **ACVR2** ]  like kinase 2  [ **ACVR1** ]  decreased.  Ref: 11675415 J Am Soc Nephrol, 2001
- BMPR I group and those of activin receptor  [ **ACVR2** ]  like kinase ALK 1 and ALK 2  [ **ACVR1** ]  ALK 1 group induced alkaline phosphatase activity in C2C12 cells.  Ref: 10564272 Mol Biol Cell, 1999
- This study demonstrated that ActR I  [ **ACVR1** ] , ActR 1B and ActR II  [ **ACVR2** ]  are expressed on human fetal lung fibroblast.  Ref: 11261590 Life Sci, 2000
